# MicroRNA 141 is associated to outcome and aggressive tumor characteristics in prostate cancer

**DOI:** 10.1038/s41598-018-36854-7

**Published:** 2019-01-23

**Authors:** Elin Richardsen, Sigve Andersen, Christian Melbø-Jørgensen, Mehrdad Rakaee, Nora Ness, Samer Al-Saad, Yngve Nordby, Mona I. Pedersen, Tom Dønnem, Roy M. Bremnes, Lill-Tove Busund

**Affiliations:** 1 Translational Cancer Research Group, Institute of Medical Biology, UiT The Arctic University of Norway, 9037 Tromsø, Norway; 2 Department of Clinical Pathology, University Hospital of North Norway, 9038, Tromsø, Norway; 3 Translational Cancer Research Group, Institute of Clinical Medicine, UiT The Arctic University of Norway, 9037 Tromsø, Norway; 4 Department of Oncology, University Hospital of North Norway, 9038 Tromsø, Norway; 5 NORMENT, Institute of Clinical Medicine, University of Oslo, Oslo, Norway; 6 Department of Urology, University Hospital of North Norway, 9038 Tromsø, Norway

## Abstract

A large number of miRNAs influence key cellular processes involved in prostate tumorigenesis. Previous studies have demonstrated high expression of miRNAs in human prostate cancer (PC) tissues and cell lines. In previous microarray data, we found miR-141 to be upregulated and miR-145 to be downregulated in PC. In this large PC cohort (n = 535), we explored the prognostic role of miR-141 and miR-145 in PC. Tumor epithelial (TE) and tumor stromal (TS) areas were evaluated separately and combined (TE + TS). *In situ* hybridization was used to evaluate the expression of the miRNAs. We found that miR-141 (TE) correlated significantly to Gleason score ≥8 (p = 0.040) and large tumor size (≥20 mm, p = 0.025) and miR-141 (TE + TS) to Gleason grade (p = 0.001). MiR-145 correlated to pT-stage (p = 0.038), tumor size (p = 0.025), Gleason grade (p = 0.051) and PSA (p = 0.032). In univariate analysis miR-141 (TE + TS) was significantly associated with biochemical failure-free survival (BFFS, p = 0.007) and clinical failure-free survival (CFFS, p = 0.021). For miR-145, there were no differences between patients with high versus low expression. In multivariate analysis overexpression of miR-141 in tumor epithelium and tumor stroma was significantly associated with BFFS (HR = 1.07 CI95% 1.00–1.14, p = 0.007). To conclude, high expression of miR-141 appears associated with increased risk of biochemical PC recurrence.

## Introduction

Prostate cancer (PC) is one of the leading causes of death among men in developed countries, but disease outcome is difficult to predict^1^. Over the last 30 years there has been a 25-fold increased number of radical prostatectomies (RP), which is consistent with the observed incidence increase of PC. This increase is mainly related to overdiagnosis due to PSA testing^2^. Recent studies have indicated that PSA concentration is unable to differentiate between indolent PC or life-threatening cancers at time of diagnosis^3^. Despite numerous of studies on prognostic- and predictive biomarkers, there is still an urgent need for more accurate stratification of aggressive versus indolent disease.

The involvement of miRNAs in gene regulatory processes and their implications in several solid cancers, including PC, make them attractive candidates for refining diagnosis, prognosis, and treatment options. miRNAs are a class of small noncoding RNA molecules that post-transcriptionally modulate gene expression by binding to the 3’- untranslated region (3′-UTR) of the target mRNA, and induce silencing of mRNA by the Argonaut (Ago) protein in the RNA-induced Silencing protein complex (RISC)^4^. Individual miRNAs are often deregulated in cancer since they are located in regions of the genome that are commonly overexpressed or deleted^5^. miRNAs are mediated by gene signaling such as deletions, amplifications, mutations, and epigenetic alterations of DNA. As a result, miRNAs can affect the synthesis of proteins necessary for tumorigenesis, disease progression, and metastasis^4,5^.

Today, there is a considerable data indicating that several miRNAs and their targets are abnormally expressed in PC^6,7^. This, alters a large range of cellular processes, including apoptosis-avoidance, cell proliferation, migration and the androgen signaling pathways^8,9^. Circulating miR-141 is consistently up-regulated in PC compared with healthy controls^10,11^ and has been suggested as biomarker for biochemical failure and clinical outcome^12,13^. In treated and untreated LnCaP cells *in vitro* and in PC xenografts in intact and castrated mice, miR-141 had the greatest androgen-dependent expression^14^. Studies of prostate tumor epithelial and adjacent stromal cells have shown that miR-141 expression was restricted to the epithelium^13^. Despite numerous studies on biogenesis and mechanisms of miR-141 in PC pathogenesis^10–14^, the accurate expression and mechanistic function is largely unclear.

miR-145 is down-regulated in metastatic PC tissue^15^. miR-145 is assumed to play a beneficial role in epithelial-mesenchymal transition (EMT) by suppression of mesenchymal markers (fibronectin) and up-regulation of the epithelial marker E-cadherin^6,16^.

From our previous microarray screening study in a limited number of human PC tissues, we found 600 of 1435 miRNAs to be highly expressed. Of these, the 50 miRNAs with the highest standard deviation (SD) were further analyzed, and the seven most up- or downregulated, which included miR-141 and miR-145, were validated by RT-qPCR^17^.

In the present study, we report the expression of mir-141 and miR-145 in TE cells and TS areas in human prostatectomy specimens and their impact on biochemical failure free survival (BFFS), clinical failure-free survival (CFFS) and prostate cancer death (PCD).

## Materials and Methods

### Patient characteristics

All radical prostatectomy samples were collected from archives of the Departments of Pathology in two different health regions in Norway (1995–2005), Northern Norway and Central Norway regional authorities. 671 patients were included, of these were 136 excluded due to pelvic radiotherapy prior surgery, previous non-superficial cancer within 5 years of PC diagnosis, lack of follow-up data and inadequate paraffin-embedded tissue blocks. The final study cohort consisted of 535 patients with complete follow-up data. Median age at surgery was 62 (47–75) years, median PSA was 8.8 (range 0.7–104.0) ng/ml, and median tumor size was 20 (range 2.0–50) mm. At last follow-up (Dec 2016) 37% had experienced BF, 11% CF and 3.4% PCD. None of the patients received pre-operative hormonal therapy. Follow-up time was defined from the date of surgery until November 31, 2015, with time of biochemical failure (BF), clinical failure (CF) and prostate cancer death (PCD) as endpoints. BF was characterized as PSA ≥ 0.4 ng/ml rising in a minimum of two different blood samples postoperatively. BF-free survival (BFFS) was the time from surgery to PSA threshold. CF was defined as local symptomatic progression and/or metastasis to bone, visceral organs or lymph nodes on CT, MR, bone scan or ultrasonography. CF-free survival (CFFS) was the time from surgery to CF.

For more extensive information regarding patients, exclusion, definitions of variables and endpoints see our previous report^18^. The tumors were re-graded in 2018 according to the updated WHO guidelines^19,20^.

### Tissue microarray construction

Tissue microarray (TMA) was used for the analyses. The cases were histologically reviewed by one uropathologist (ER) and the most representative areas of epithelial tumor cells and adjacent tumor stroma were selected for the donor block. The TMAs were made by using a tissue-arraying instrument (Beecher Instruments, Silver Springs, MD). The detailed methodology has been reported previously^21^. A 0.6 mm stylet was used to sample. Core samples from two different areas of neoplastic epithelial tumor cells and two of adjacent tumor stroma were collected. Prostate cores from 20 patients without any history of malignancy were used as negative controls. The core samples were inserted into an empty recipient paraffin block according to a predefined coordinate pattern.

### *In situ* hybridization (ISH)

Chromogen *in situ* hybridization (cISH) was performed both manual and automatic. We used labelled locked nucleic acid (LNA) modified probes from Exiqon; miR-141 (hsa-miR-141–3p MIMAT0000432, miRCURY LNA Prod. No. 38042-15) and miR-145 (hsa-miR-145-5p MIMAT0000437, miRCURY LNA Prod. No. 88068-15). The Positive control (U6 hsa/mmu/rno, No 99002-15) consolidate good sensitivity of the method with distinct and strong staining. As negative control, we used the scrambled-miRNA (No. 99004-15). As positive and negative tissue control, we used a multi TMA-block, which included 12 different organs with normal and tumor tissue. RNAse-free water was used during sectioning and as diluent agent for buffer solutions. 4 µm TMA slides was attached to Super Frost Plus slides by overnight heating (60 °C).

MANUAL protocol: Sections were deparaffinized in xylene (3 × 10 min) and hydrated in ethanol solutions to PBS (pH 7.4). Proteinase-K 20 µg/ml treatment were done in PK-buffer (5 mM Tris-HCL, pH 7.5, 1 mM NaCl, autoclaved) at 37 °C for 20 min in a ThermoBrite hybridizer. After this treatment, TMAs were rehydrated through ethanol and air-dried. The LNA-probes were denatured by heating to 90 °C for 4 min.

Hybridization of the LNA-probes miR-141 (25 nM), scrambled miRNA (50 nM) and U6 (2 nM) was done by using a ThermoBrite hybridizer (50 °C for 60 min). Washes was done in room temperature by using 5 x SSC buffer, pre-heated SSC buffers (50 °C): 5 min in 5 x SSC, 2 × 5 min in 1 x SSC, 2 × 5 min in 0.2 SSC, and 5 min in RT 0.2 x SSC. The blocking solution was DIG wash (11 093 274 910, Roche, Mannheim, Germany) and Block buffer set (11 585 762 001, Roche, Mannheim, Germany) for 15 min in a humidity chamber. Alkaline phosphatase (AP)-conjugated anti DIG 1:800 was incubated for 30 min at 30 °C in a humidity chamber for immunologic detection. After PBS-T wash the substrate enzymatic reactions was carried out with NBT/BCIP (11 697 471 001, Roche, Mannheim, Germany) at 30 °C in the ThermoBrite for 120 min. To stop the reaction, we used KTBT buffer (50 nM Tris-Hcl, 150 nM NaCl, 10NM KCI) for 2 × 5 min, followed by wash in double distilled water.

Counterstaining of section was done by use of nuclear fast red (WALDECK, ZE-012-250) at room temperature before water tap rinse. The last step was the dehydration by ethanol at increasing gradients and mounting of the Histokitt mounting medium (Assistant-Histokitt, 1025/250 Sondheim/Rhoen Germany).

AUTOMATIC protocol in Discovery Ultra: For miR145, sections were deparaffinized in EZ Prep (Roche 950-100) at 68 °C (3 × 12 min). Pretreatment heating was done at 95 °C with CC1 (Roche 950-500), for hsa-miR-145 (32 min), and scrambled miRNA (24 min). For U6, we used a combination of heat mediated and enzymatic pretreatment (CC1 for 8 min at 95 °C, Protease III, Roche 760-2020 for 16 min at 37 °C). After pretreatment, the sections were rinsed by using Reaction Buffer (Roche 750-300), followed by RiboWash, SSPE (Roche 760-105). The probes were diluted in 1:1 Exiqon microRNA ISH buffer (product No.9000) and Elix RNAse free water. MiR-145 (50 nM), scramble miRNA (10 nM) and U6 (0.5 nM) were applied and denaturation was 8 min at 90 °C. Hybridization in 60 min was done for Mir-145 (50 °C), scrambled MiRNA (57 °C) and U6 (55 °C). We washed with 2.0X RiboWash, SSPE buffer for 2 × 8 min and used the same temperatures as for the hybridization procedure. After rinsing with Reaction Buffer, sections were blocked against unspecific binding for 12 min at 37 °C (from DIG wash and Block buffer set, 11 585 762 001, Roche, Mannheim, Germany).

For the immunologic detection, we used prediluted Alkaline phosphatase (AP)-conjugated anti DIG (Anti-DIG-AP, Roche 760-4825) for 20 min at 37 °C. The sections were rinsed with Reaction Buffer and EZ Prep before the substrate enzymatic reactions for 20 min at 37 °C (NBT/BCIP CromoMap Blue kit, Roche 760-161). The sections were rinsed once more with Reaction Buffer and counterstained for 4 min with Red Stain II (Roche 780-2218). The last step was the dehydration process, which was done by using ethanol solutions and mounted with Histokitt mounting medium (Assistant-Histokitt, 1025/250 Sondheim/Rhoen Germany).

To ensure good distribution of reagents and to protect section from drying, all incubations were added LCS (Liquid Coverslip oil, Roche 650-010).

Data availability. The datasets generated during and or analyzed during the current study are available from the corresponding author on reasonable request.

### Scoring of *in situ* hybridization

We used the ARIOL imaging system (Genetix, San Jose, USA) in the scoring process of the TMAs. The TMAs were scanned at low resolution (1.25x) and high resolution (20x) by using Olympus BX 61 microscope. We scored, semi-quantitatively, TE and TS separately. Both intensity and density were scored. Intensity scoring scale: 0 = negative, 1 = weak, 2 = moderate, 3 = strong. Density was scored as follows: percentage positive cells examined in TE or TS was scored by using the following system: 0 = 0%, 1 = ≤5%, 2 = 5–50%, 3 ≥50%, then a mean score was calculated. The scoring values were dichotomized into high or low intensity. In both TE and TS areas, the cut-off was defined as the density level × 4^th^ quartile. High co-expression (TE + TS) of miR-141 and miR-145 was defined as low expression (≤1) and high expression (≥2). The samples were anonymized and independently scored (ER/CMJ and ER/SAS). In case of disagreement, the slides were re-examined until a final consensus was reached.

### Statistical methods

The IBM SPSS, version 24 (SPSS Inc., Chicago, IL, USA) was used for statistical analyses. Wilcoxon signed rank test was used to assess differences in miR-141 and miR-145 expression between normal tissue and cancer tissue. Spearman’s Correlation coefficient was used for correlation analysis between miR-141 and miR-145 expression and clinicopathological markers. The Kaplan-Meyer method was used for drawing survival plots for BF and CF, and statistical differences was done by using log-rank test. Variables from the univariate analyses with a p < 0.05 were included in a multivariate survival analysis by using a backward stepwise multivariate Cox regression model with a probability for stepwise entry removal at 0.05 and 0.10, respectively. The significance level was set to p-value <0.05.

### Ethics

The current study was approved by the Regional Committee for Medical and Health Research Ethics, REK Nord (Project Application 2009/1393), including a mandatory re-application January 22. 2016. Due to the retrospective study design, the tissue blocks were collected from 1995 and 2005, and many patients were deceased, REK Nord considered written consent not to be necessary. In addition, the Data Protection Official for Research and The National Data Inspection Board approved the establishment of the database. The patient records were anonymized prior to the analyses. The reporting of clinicopathological variables, survival data and biomarker expressions was conducted in accordance with the REMARK guidelines.

## Results

### Patient characteristics

Patients’ characteristics are presented in Table 1. Median age at surgery was 62 (47–75) years, median PSA was 8.8 (range 0.7–104.0) ng/ml, and median tumor size was 20 mm (range 2.0–50). The prostatectomies specimens were retropubic in 435 and perineal in 100 cases. At the end of follow-up, 200 (37%) patients had BF, 56 (11%) had CF, and 18 (3%) were dead of PC.

**Table 1:** Summary of patient characteristics, clinicopathological variables, miR-141 and miR-145. Univariate analyses; log-rank test.

Characteristic	Patients N (%)	BF (200 events)		CF (56 events)		PCD (18 events)	
		5-year EFS (%)	P	10-year EFS (%)	P	10-year EFS (%)	P
**Age**			NS		**0.008**		NS
≤65 years	357 (67)	76		92		97	
>65 years	178 (33)	70		88		96	
**pT-stage**			**<0.001**		**<0.001**		**<0.001**
pT2	374 (70)	83		96		98	
pT3a	114 (21)	60		86		98	
pT3b	47 (9)	43		73		89	
**Preoperative PSA**			**<0.001**		NS		**<0.001**
PSA < 10	308 (57)	81		93		99	
PSA > 10	221 (42)	68		88		95	
Missing	6 (1)						
**Gleason Grade Group**			**<0.001**		**<0.001**		**<0.001**
1 (3 + 3)	183 (34)	83		98		99	
2 (3 + 4)	220 (41)	77		93		98	
3 (4 + 3)	8 (15)	70		84		95	
4 (4 + 4)	19 (4)	59		76		94	
5 (≥9)	33 (6)	37		67		87	
**Tumor Size**			**<0.001**		**0.019**		NS
0–20 mm	250 (47)	83		94		99	
>20 mm	285 (53)	68		88		96	
**PNI**			**<0.001**		**<0.001**		**0.002**
No	401 (75)	83		95		98	
Yes	134 (25)	68		81		93	
**PSM**			**0.049**		NS		NS
No	249 (47)	81		94		97	
Yes	286 (53)	69		89		97	
**Non-apical PSM**			**<0.001**		**<0.001**		**0.022**
No	381 (71)	82		95		98	
Yes	154 (29)	57		81		94	
**Apical PSM**			NS		NS		NS
No	325 (61)	74		90		96	
Yes	210 (39)	77		92		98	
**Vascular infiltration**			**<0.001**		**<0.001**		**<0.001**
No	492 (92)	77		93		98	
Yes	43 (8)	47		71		88	
**Surgical procedure**			NS		NS		NS
Retropubic	435 (81)	77		90		97	
Perineal	100 (19)	68		95		98	
**miR-141** (**TE** + **TS**)			**0.007**		**0.021**		NS
High	299	126		38		11	
Low	164	50		10		5	
**miR-145** (**TE** + **TS**)			NS		NS		NS
High	247	91		27		10	
Low	248	97		25		7	

Abbreviations: BF = biochemical failure; EFS = event free survival, CF = clinical failure. PCD = prostate cancer death; P = p-value; PNI = perineural infiltration; PSM = positive surgical margin; NS = not significant.

### Expression of miR-141 and miR- 145 and their correlations to clinicopathological variables

**Figure 1:**
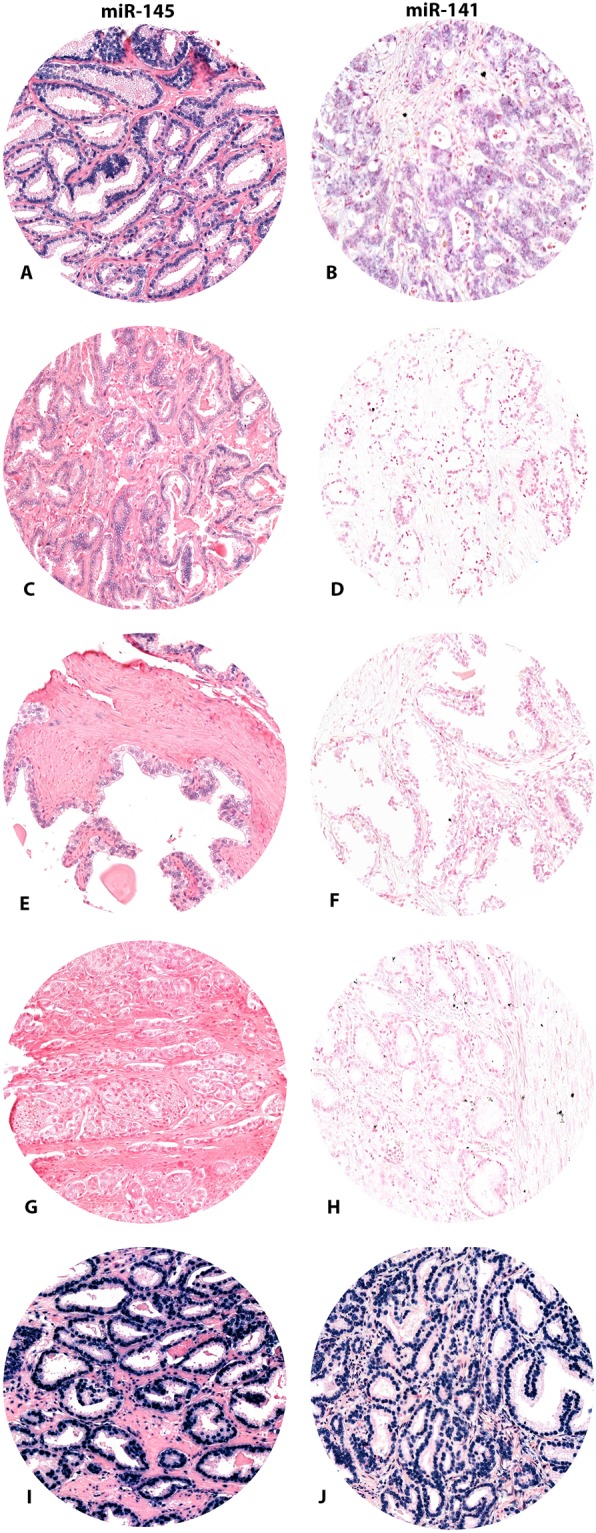
Immunohistochemical staining of (**A**) miR-145 and (**B**) miR-141. High expression of mir-145 (**A**) and miR-141 (**B**). Low expression of miR-145 (**C**) and miR-141 (**D**). Normal prostate tissue, miR-145 (**E**) and miR-141. Scrambled miR-145 (**G**), miR-141 (**H**) and U6, miR-145 (**I**) and miR-141 (**J**).

Of the total cohort, for miR-145 we had 495 cores available for miR-145 scoring, 248 with low expression and 247 with high expression. miR-145 was weaker in normal epithelial- and stromal cells compared to cancer tissue. Its staining pattern was nuclear and mainly in TE (Fig. 1A,C,E). For miR-141 we had 463 cores available for scoring, 164 cores with low expression and 299 cores with high expression. Overall, the expression of miR-141 was higher in tumor tissue compared with normal prostatic tissue, and mainly in TE (Fig. 1B,D,F). The correlation between the miRs expression and clinicopathological variables were weak or non-significant *(r* = <0.2). MiR-141 in TE correlated significantly to Gleason score (p = 0.040) and large tumor size (p = 0.050), and in TE + TS to Gleason score (p = 0.001). MiR 145 (TE + TS) correlated to pT-stage (p = 0.038), tumor size (p = 0.025), Gleason score (p = 0.051) and pre-operative PSA (p = 0.032).

### Univariate analyses

**Figure 2:**
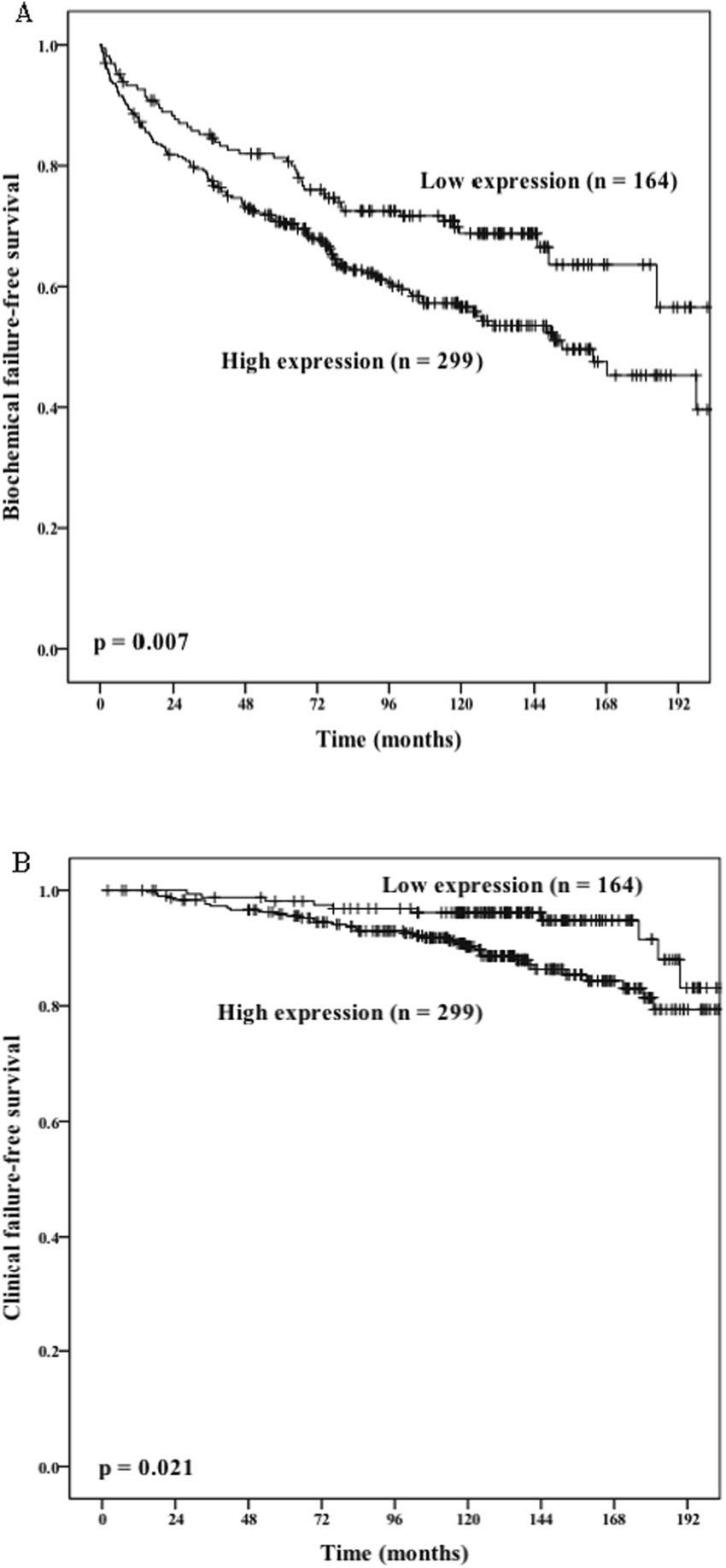
miR-141 (TE + TS). Patients cohort were dichotomised into low and high-risk groups evaluated by using Kaplan-Meier analysis of, (**A**) Biochemical failure-free survival. (**B**) Clinical failure-free survival. The p-value for a two-sided log rank test is shown for both.

PC patients with high co-expression (TE + TS) of miR-141 had significantly lower BFFS, Fig. 2A (p = 0.007) and CFFS, Fig. 2B (p = 0.021) than patients with low expression. Analyzing mir-141 separately in TE and TS, only TE was significant for BFFS (p = 0.045), but not for CFFS (p = 0.746). The clinicopathological variables which were associated with BFFS, CFFS and PCDFS are listed in Tables 1 and 2. Shortly, the following was associated with BFFS; pT-stage, preoperative PSA, Gleason grade, tumor size, perineural infiltration (PNI), positive surgical margin (PSM), non-apical PSM and vascular infiltration (VI). Association with CFFS; pT-stage, Gleason grade, tumor size, PNI, PSM, and VI. For PCD; pT-stage, preoperative PSA, Gleason grade, PNI, non-apical PSM and VI.

**Table 2:** High expression of miR-141 is a prognostic factor in prostate cancer of 535 patients.

Characteristics	BFFS			CFFS		
	HR	CI 95%	p	HR	CI 95%	p
**Age**			NS			**0.024**
≤65				1		
>65				0.049	0.27–0.91	
**PT-stage**			<**0.001**			NS
pT2	1					
pT3a	0.31	0.21–0.56	<**0.001**			
pT3b	0.57	0.36–0.90	**0.017**			
**Gleason Grade Group**			**0.036**			**0.005**
1 (3 + 3)	1			1		
2 (3 + 4)	0.54	0.30–0.98	**0.043**	0.10	0.03–0.34	<**0.000**
3 (4 + 3)	0.72	0.41–1.26	0.250	0.42	0.19–1.00	**0.039**
4 (4 + 4)	1.02	0.57–1.84	0.947	0.56	0.23–1.35	0.195
5 (≥9)	2.53	1.51–4.27	0.604	0.38	0.08–1.71	0.207
**PSM**			**0.002**			NS
No	1					
Yes	0.59	0.42–0.82				
**Apical PSM**			**0.033**			NS
No	1					
Yes	1.44	1.03–2.00				
**PNI**			**0.011**			**0.001**
No	1			1		
Yes	0.65	0.47–0.91		0.17	0.06–0.50	
**miR-141** (**TE** + **TS**)			**0.050**			NS
Low	1					
High	1.07	1.00–1.14				

Multivariate analyses; Cox regression with backward conditional. Abbreviations: BFFS = biochemical failure-free survival, CFFS = clinical failure-free survival. PSM = positive surgical margins; PNI = perineural infiltration; TE = tumor epithelium; TS = tumor stroma areas.

For miR-145, there were no association with BFFS (p = 0.348), CFFS (p = 0.895) or PCDFS (p = 0.520) when assessed in patients with high versus low expression. When analyzing TE and TS as separate compartments, or combined (TE + TS) no association were found for miR-145.

### Multivariate analyses

In the multivariate analyses (Table 2), high expression of miR-141 (TE + TS) was borderline significant for BFFS (HR = 1.07 95% CI 1.00–1.14, p = 0.050), not with CFFS (HR = 2.32 95% CI 0.94–5.73, p = 0.068). In addition, the following clinicopathological variables were significant predictors for BFFS: pT-stage (p < 0.001), Gleason score (p = 0.036), PSM (p = 0.002), non-apical PSM (p = 0.003), apical PSM (p = 0.033) and PNI (p = 0.011). CFFS, age (p = 0.024), Gleason grade group (p = 0.005), and for PCDFS, only VI (p = 0.002) and PNI (HR = 0.17, 95% CI 0.06–0.50, p = 0.001).

## Discussion

In this large cohort of 535 radical prostatectomy specimens we found that the expression of miR-141 was associated with an increased risk of BFFS and CFFS from PC. Both by analyzing the tumor tissue as one compartment (TE + TS) or only in the TE compartment we found the same association for increased risk of biochemical- and clinical failure. Nevertheless, which role the various miRNAs play at different stages of PC progression and how their expression change during the multistep carcinogenesis, is poorly understood.

We found that miR-141 was mainly expressed in the TE, which is consistent with cell line studies^12,14^. Most studies on miR-141 are, however, from liquid biopsies such as urine, serum, plasma and whole blood. Mitchell *et al*.^22^. reported that tumor-derived miRNAs can enter the circulatory system and be measured in serum and plasma as important blood-based biomarkers of human cancer. The authors showed that circulating miR-141 is significantly elevated in the sera of PC patients compared to healthy controls^22^. Brase *et al*.^12^. reported that high levels of circulating miR-141 were associated with more aggressive and advanced disease (high Gleason score and lymph node metastases), Waltering *et al*. showed that miR-141 up-regulation in plasma of metastatic PC patients as well as in cell lines after castration, and that this up-regulation induced growth of LNCaP cells^14^. This may imply that miR-141 regulate androgen, which plays a crucial role in the growth of both androgen-dependent and castration-resistant PC. Besides, Agaoglu *et al*.^11^. reported a significantly higher circulating miR-141 in patients with locally advanced-stage disease. Measured in liquids (blood and serum), miR-141 seems to be one of the more promising markers for PC progression^11–13^.

There is mounting evidence that the androgen receptor (AR) is not the only effective endocrine receptor in this complex process^14^. Previous studies suggest involvement of both the glucocorticoid-, estrogen- and progesterone receptors^23–25^. One study by Larne *et al*. found that miR-145, by suppressing the AR in PC cells, correlated to PC prognosis^26^. Their results were verified in clinical prostate specimens by demonstrating inverse correlations between miR-145 and AR expression as well as serum PSA levels. In addition, miR-145 was found to regulate androgen-dependent cell growth *in vitro*. IHC studies on PR expression in PC have demonstrated contradicting results and only a few reports have addressed the roles PR’s plays in prostate carcinogenesis. We have previously demonstrated a general distribution of PR in tumor epithelium of PC^27^. The present findings of a correlation between miR-141 expression in PC tumor tissue and PR suggest that mRNAs may also be involved in PR regulation or vice versa.

We found that miR-145 was weakly expressed in both tumor tissue and normal prostatic tissue and was correlated to clinicopathological variables associated with worse outcome. In spite of this, miR-145 was not associated with biochemical- or clinical failure. Our findings are in line with Pang *et al*. and Schaefer *et al*. failing to detect any associations between miR-145 and clinicopathological variables^6,28^. However, other studies have reported that loss of the tumor suppressing properties of miR-145 is correlated with a higher Gleason grade, bone metastases and shorter disease-free survival^29,30^. Downregulation of miR-145 may lead to enhanced cell proliferation, migration, and invasion in PC^29^. Fuse *et al*. also demonstrated that the capacity of PC3 and DU145 PC cells lines to proliferate, migrate, and invade was impaired by transfection with miR-145^31^. miR-145 may inhibit PC cell proliferation by targeting Fasin homolog 1 (FSCN1), an actin bundling protein, which is involved in cell motility, adhesion and cellular interactions during tumorigenesis and metastasis^31^.

Some of the innate properties of miRNAs make them attractive as potential biomarkers, as they may can be isolated from most body fluids and easily detected in small volume samples. However, their profile is not organ-specific and discrimination between different types of malignancies is not yet possible^32,33^. An ideal biomarker of tumors, measured in liquid biopsies or in FFPE, should be specific, sensitive, and proportional to tumor load. Studies have demonstrated that for many of the miRNAs, the circulating miRNAs and tissue miRNAs are comparable. High tumoral association of miRNAs in liquid biopsies is not necessarily equal to the level expressed at protein level when measured by tumor tissue immunohistochemistry. Moreover, FFPE is an extremely powerful preservation agent. Therefore, once a tissue specimen is fixed, it can be stored at room temperature for years, unlike current preservation properties of miR’s.

Possible limitation of our study includes its retrospective nature as well as the use of old tissue blocks, which could have affected the results of hematoxylin/eosin and immunohistochemical staining. However, fresh sections were cut and stained for best results. To the best of our knowledge this is the largest study visualizing miR-141 and mir-145 on hormone naïve PC tissue by using IHC.

## Conclusions

We found that high expression of miR-141 to be significantly associated to worse PC outcome. In TE a high expression was associated with BFFS and in TE + TS was associated with a higher risk of BFFS and CFFS. We also found that miR-145 correlated with more aggressive features of prostate cancer. This knowledge may be valuable for further studies, which should provide further mechanistical explanation for the role of miR-145 in PC, in particular regarding target genes of the miR in PC.
